# Medical Statistics

**Published:** 1852-05

**Authors:** 


*Medical Statistics.*

| Colleges. | Matriculants. | Graduates. |
|---|---|---|
| Jefferson Med. College, - - - | 506 | 227 |
| Med. College of Ohio, - - - - - | 115 | 44 |
| Starling Med. Col., - - - - - | 146 | 35 |
| Kentucky School of Medicine, - - | 112 | 27 |
| Rush Med. College, - - - - - | 120 | 37 |
| Medical Department of the University of La., | 186 | 42 |
| Med. School of Harvard University, - | 126 | 38 |
| Med. College of the University of Nashville, | 121 | 33 |
| College of Physicians and Surgeons, N. Y., | 197 | 59 |
| University of the City of N. Y., - - | 179 | 98 |
| Med. College of Georgia, - - - - | 158 | 50 |
| Med. Department of the University of Mich., | 159 | 27 |
| Med. College of the State of S. C., - | 232 | 103 |
| Med. Institution of Yale College, - - | | 14 |
| University of Louisville, - - - - | 261 | |
| N. Y. Med. College, - - - - - - | 69 | |
| N. H. Med. College, - - - - - | | 16 |
| Female Med. College of Philadelphia, - | | 8 |
| University of Buffalo, - - - - - | | 20 |
| Med. School of Maine, - - - - | 60 | |
| University of Pennsylvania, - - - | | 157 |

